# First, Do No Harm (Gone Wrong): Total-Scale Analysis of Medical Errors Scientific Literature

**DOI:** 10.3389/fpubh.2020.558913

**Published:** 2020-10-16

**Authors:** Atanas G. Atanasov, Andy Wai Kan Yeung, Elisabeth Klager, Fabian Eibensteiner, Eva Schaden, Maria Kletecka-Pulker, Harald Willschke

**Affiliations:** ^1^Ludwig Boltzmann Institute for Digital Health and Patient Safety (LBIDHPS), Medical University of Vienna, Vienna, Austria; ^2^Institute of Genetics and Animal Biotechnology of the Polish Academy of Sciences, Magdalenka, Poland; ^3^Institute of Neurobiology, Bulgarian Academy of Sciences, Sofia, Bulgaria; ^4^Department of Pharmacognosy, University of Vienna, Vienna, Austria; ^5^Oral and Maxillofacial Radiology, Applied Oral Sciences and Community Dental Care, Faculty of Dentistry, The University of Hong Kong, Hong Kong, China; ^6^Division of Pediatric Nephrology and Gastroenterology, Department of Pediatrics and Adolescent Medicine, Comprehensive Center for Pediatrics, Medical University of Vienna, Vienna, Austria; ^7^Department of Anaesthesia, Intensive Care Medicine and Pain Medicine, Medical University Vienna, Vienna, Austria

**Keywords:** medical errors, bibliometric analysis, adverse drug events, patient safety, public health

## Abstract

**Objective:** Medical errors represent a leading cause of patient morbidity and mortality. The aim of this study was to quantitatively analyze the existing scientific literature on medical errors in order to gain new insights in this important medical research area.

**Study Design:** Web of Science database was used to identify relevant publications, and bibliometric analysis was performed to quantitatively analyze the identified articles for prevailing research themes, contributing journals, institutions, countries, authors, and citation performance.

**Results:** In total, 12,415 publications concerning medical errors were identified and quantitatively analyzed. The overall ratio of original research articles to reviews was 8.1:1, and temporal subset analysis revealed that the share of original research articles has been increasing over time. The United States contributed to nearly half (46.4%) of the total publications, and 8 of the top 10 most productive institutions were from the United States, with the remaining 2 located in Canada and the United Kingdom. Prevailing (frequently mentioned) and highly impactful (frequently cited) themes were errors related to drugs/medications, applications related to medicinal information technology, errors related to critical/intensive care units, to children, and mental conditions associated with medical errors (burnout, depression).

**Conclusions:** The high prevalence of medical errors revealed from the existing literature indicates the high importance of future work invested in preventive approaches. Digital health technology applications are perceived to be of great promise to counteract medical errors, and further effort should be focused to study their optimal implementation in all medical areas, with special emphasis on critical areas such as intensive care and pediatric units.

## Introduction

Medical errors are a leading cause of patient morbidity and mortality. Recent mortality analysis in the United States ranked medical errors as the third major cause of death, following heart disease and cancer, which were ranked on the first and second place, respectively (1). A recent meta-analysis of 70 studies involving a total of 337,025 patients revealed that the average rate of preventable patient harm was 6%, of which 12% was severe or led to death (2). The same study also revealed that errors related to drugs (25%) and other treatments (24%) were the largest sources of preventable patient harm, and incidents were more likely to occur in advanced specialties (intensive care or surgery) in comparison to general hospitals (2).

Aside from patient harms and suffering, medical errors contribute to adverse mental and emotional effects on patient relatives and involved healthcare providers (3). Moreover, medical errors result in significant economic burden due to additional healthcare costs and lost productivity from missed workdays (4).

While sometimes medical errors have been scientifically reviewed and specifically examined in relation to the presence of adverse patient outcomes or injury, a more general definition is not linked to outcomes. Upon systematically examining this research area, Grober and Bohnen have proposed a more extensive definition of medical errors being “an act of omission or commission in planning or execution that contributes or could contribute to an unintended result” (5).

Better understanding of medical errors can be of a great importance because it may yield approaches aiming at their reduction. In this context, systematic analysis of the existing scientific literature in that area is of potentially high significance. Bibliometric analysis is a versatile approach involving evaluation of different parameters related to published literature, and its application can yield quantitative information reveling, e.g., prevailing research themes, characteristics and temporal trends (globally or within a specific scientific area), and impactful research studies, authors, and institutions (based on citation analysis) (6–9). Applied to the medical errors research literature, such insights can be of high value for both researchers and non-experts for rapid orientation and navigation within this scientific field and for the identification of relevant topics, trends, experts, and potential collaborator-candidates. Thus, because the existing scientific literature on medical errors has not yet been evaluated utilizing a bibliometric approach on a total scale, the aim of this work was to identify and bibliometrically analyze the relevant literature in order to gain new insights into this important medical research area.

## Methods

### Data Sources

In January 2020, Web of Science (WoS) Core Collection (https://webofknowledge.com) database was queried in order to identify relevant publications concerning the targeted scientific area using the following search strategy: TOPIC = (“medic^*^ error^*^”) OR (“medic^*^ mistake^*^”) OR (“mistake^*^ in medic^*^”) OR (“error^*^ in medic^*^”) OR (“healthcare^*^ error^*^”) OR (“healthcare^*^ mistake^*^”) OR (“mistake^*^ in healthcare^*^”) OR (“error^*^ in healthcare^*^”) OR (“preventable patient harm^*^”) OR (“preventable harm^*^ in healthcare^*^”). This search strategy identified articles containing medical/healthcare (or derivatives of these words) in combination with error/mistake (or derivatives of these words, including plural forms, i.e., errors/mistakes) in the publication titles, abstracts, or keywords. No additional filters were used for the search.

### Definitions and Data Extraction

Relevant publications were defined as those fulfilling the search criteria indexed in WoS at the time of the search. For this study, countries/regions were defined as the geographic locations listed by WoS that were based on the addresses of the author's affiliations (institutions). The publication type was defined as the document classification tagged by WoS to each of the publications, e.g., article, review, etc. The WoS categories referred to the journal categories assigned to each journal by WoS, so that one journal could belong to multiple categories.

The complete data set was extracted from WoS by the “Export Records to File” function. A maximum of 500 records in “Full Record and Cited References” were exported at a time, in the file format “Tab-delimited (Mac).” The procedure was repeated until the whole data set was covered. Initial bibliographic data were extracted with the WoS “Analyze Results” and “Create Citation Report” features.

### Analysis

The 10 most productive authors, institutions, countries/regions, journals, and WoS categories were identified for the all-time data sets. The number of publications (*n*), share of the total publication of the respective period (%), citations per publication (CPP), H index (except for journals), and impact factor (only for journals) were recorded.

The bibliographic data sets were loaded into VOSviewer, a bibliometric software, to relate citation data and words appearing in the titles/abstracts for four time periods: all-time, the 1990s and before, the 2000s, and since the 2010s. Only words recurring in >1% of the publications in the respective data sets were analyzed and visualized. The relationship was similarly analyzed with author keywords recurring in >0.5% of the publications in the respective data sets. Bubble maps were generated to illustrate the changes in the literature.

## Results

**Figure 1:**
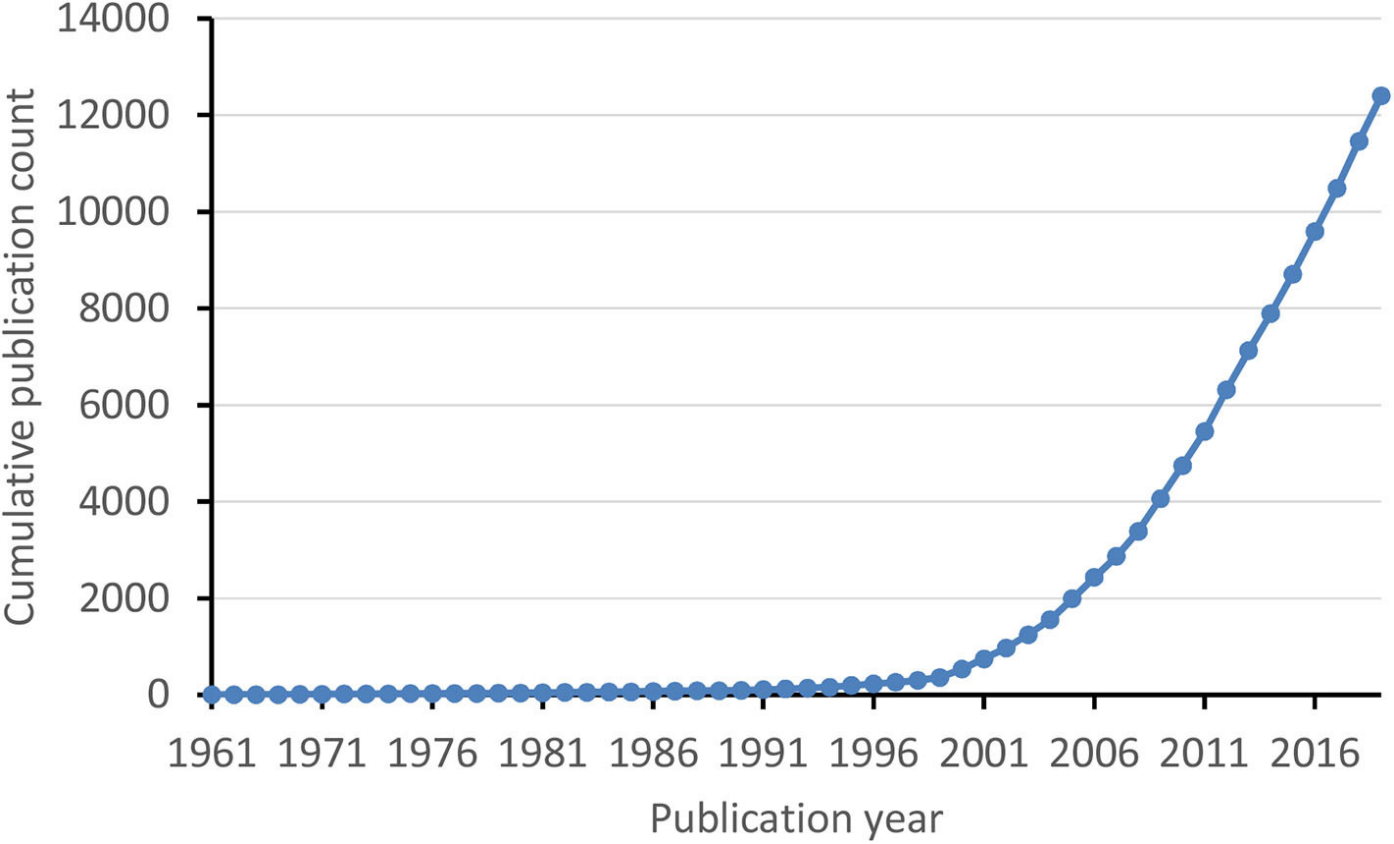
Cumulative publication count of medical errors literature over time.

**Figure 2:**
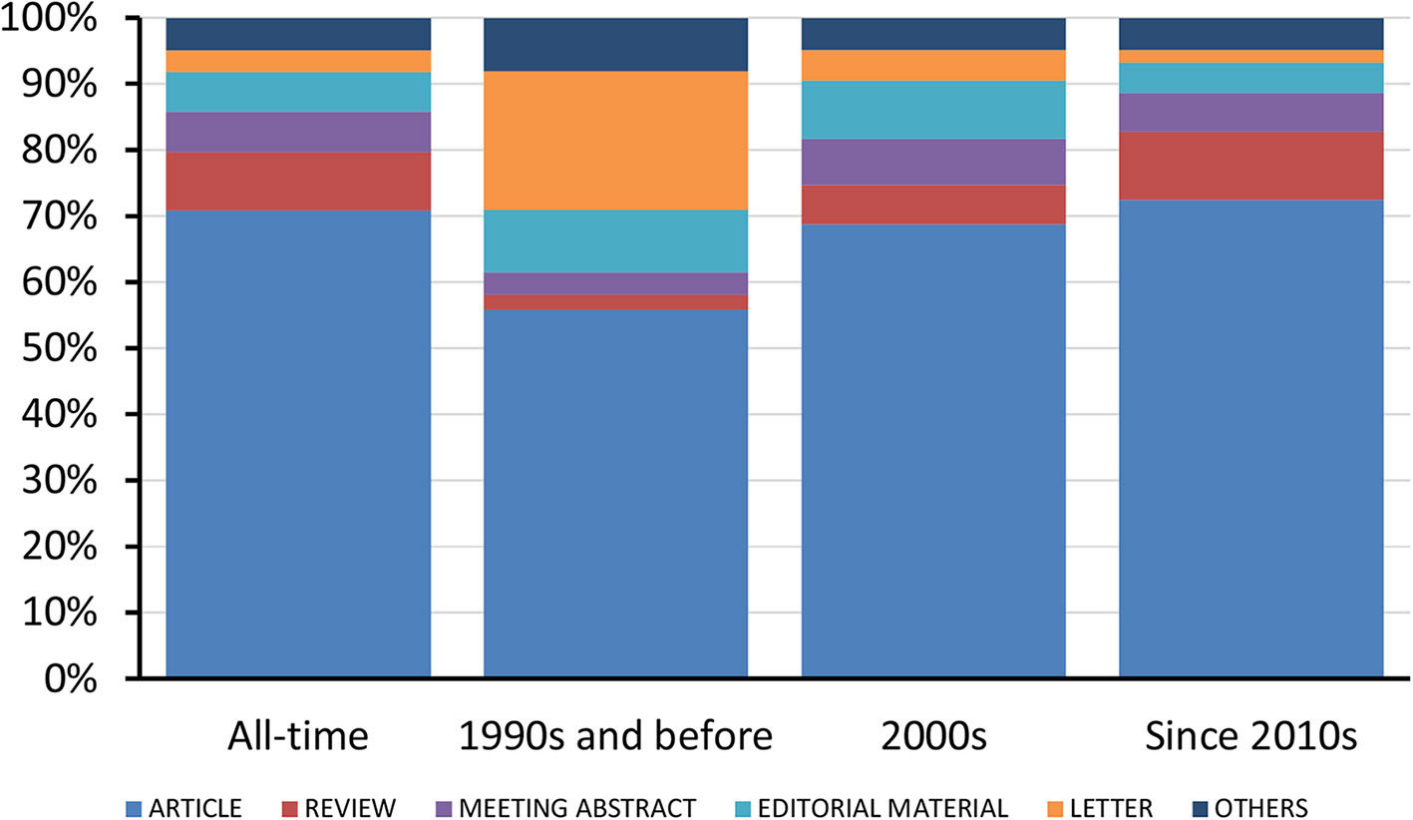
Trends in the ratio of various publication types.

In total, 12,415 publications concerning medical errors were indexed in WoS, dating back to 1961 and accumulating gradually ever since, surpassing 1,000 total publications in 2003 and 10,000 in 2017 (Figure 1). There were 358 articles published in the 1990s and before, 3,705 articles in the 2000s, and 8,352 articles since the 2010s. Overall, 70.9% of the publications were original articles and 8.8% were reviews, resulting in a ratio of 8.1:1 (Figure 2). The ratio of original articles increased from 55.9% in the 1990s and before, up to 72.4% since the 2010s. Simultaneously, the ratio of reviews also increased from 2.2 up to 10.3%, whereas the ratio of letters and editorial materials declined.

**Figure 3:**
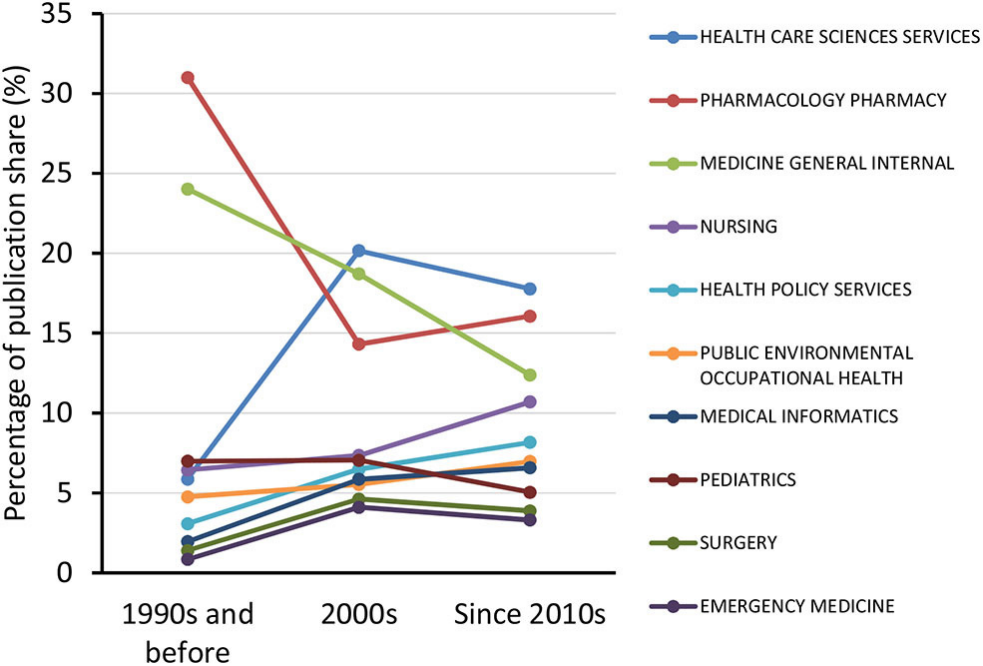
Trends in the publication share of various Web of Science journal categories.

The all-time 10 most productive authors, institutions, countries/regions, journals, and WoS journal categories are listed in Table 1. Eight of the top 10 institutions were from the United States, and one each in Canada and the United Kingdom. The United States had contributions to nearly half of the total publications. The *American Journal of Health-System Pharmacy* (impact factor 2.012) was the most productive journal. In addition, publication shares of “pharmacology pharmacy” and “medicine general internal” journals decreased over the decades, whereas the shares of “healthcare sciences services” and “nursing” journals surged (Figure 3).

**Table 1:** The all-time 10 most productive authors, institutions, countries/regions, journals, and journal categories.

	**No. of publications (*n*)**	**Share of the total publication (%)**	**Citations per publication (CPP)**	**H index**
**Author**				
Bates D.W.	182	1.5	96.1	61
Gallagher T.H.	62	0.5	39.6	25
Franklin B.D.	47	0.4	27.9	18
Landrigan C.P.	47	0.4	78.0	21
Van den Bemt P.M.L.A.	45	0.4	18.3	17
Kaushal R.	42	0.3	118.4	23
Wu A.W.	38	0.3	57.2	23
Pronovost P.J.	37	0.3	34.7	18
Rothschild J.M.	35	0.3	95.7	22
Sheikh A.	35	0.3	23.3	16
**Institution**				
Harvard University	753	6.1	53.4	92
Brigham Women's Hospital	404	3.3	65.3	74
University of California System	357	2.9	35.4	54
University of Toronto	287	2.3	31.6	46
Johns Hopkins University	244	2.0	33.4	49
University of London	204	1.6	25.9	42
University of Texas System	198	1.6	24.3	35
University of Pennsylvania	190	1.5	35.0	39
Pennsylvania Commonwealth System of Higher Education	184	1.5	25.3	37
University of Washington	183	1.5	41.7	39
**Country/region**				
United States	5,759	46.4	25.5	156
England	973	7.8	19.1	66
Canada	687	5.5	22.1	59
Australia	590	4.8	15.0	42
Germany	442	3.6	10.8	35
France	415	3.3	9.6	31
Spain	371	3.0	6.5	24
The Netherlands	344	2.8	21.9	42
Italy	280	2.3	15.3	29
Switzerland	246	2.0	17.3	34
**Journal (impact factor 2018)**				
American Journal of Health-System Pharmacy (2.012)	261	2.1	19.6	41
Journal of General Internal Medicine (4.606)	173	1.4	37.3	43
Drug Safety (3.526)	158	1.3	15.5	27
Quality & Safety in Health Care (2.160 in 2012; superseded by BMJ Quality & Safety)	144	1.2	54.3	49
International Journal of Clinical Pharmacy (1.692)	143	1.2	4.1	13
Journal of the American Medical Informatics Association (4.292)	140	1.1	57.4	41
Studies in Health Technology and Informatics (NA)	126	1.0	3.1	8
Pediatrics (5.401)	124	1.0	57.8	45
BMJ Quality & Safety (7.043)	122	1.0	23.7	27
Joint Commission Journal on Quality and Patient Safety (NA)	114	0.9	21.9	27
**WoS journal category**				
Health care sciences services	2,253	18.1	24.2	101
Pharmacology pharmacy	1,983	16.0	11.7	67
Medicine general internal	1,813	14.6	31.2	109
Nursing	1,189	9.6	12.1	53
Health policy services	933	7.5	19.7	67
Public environmental occupational health	804	6.5	13.3	48
Medical informatics	772	6.2	21.8	60
Pediatrics	707	5.7	19.5	57
Surgery	500	4.0	17.8	46
Emergency medicine	431	3.5	16.6	44

**Figure 4:**
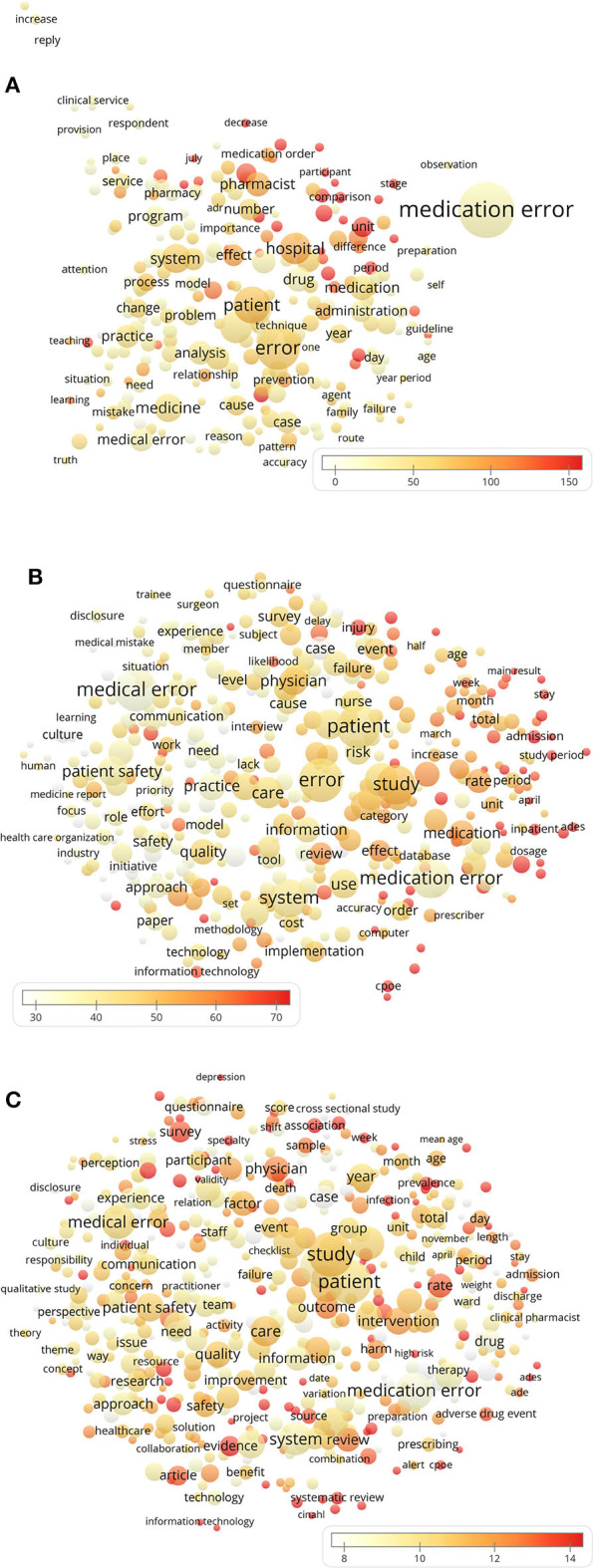
The use of terms in the titles and abstracts of the publications during **(A)** the 1990s and before, **(B)** the 2000s, and **(C)** since the 2010s. The color of the bubbles indicates the citations per publication (CPP) containing the terms; the bubble size indicates the number of publications and the distance between the bubbles indicates the frequency of co-occurrence of the terms.

The 10 most recurring and most cited terms for all-time and each time period are listed in Table 2. The medication error was one of the most frequently mentioned types of medical errors, and “adverse drug event” was the term associated with the highest citation rate of the associated publications. Together with adverse drug events (ADEs), computerized physician order entry (CPOE) and burnout (of healthcare providers) are also among the topics associated with high average citations. Bubble maps are shown in Figure 4 to illustrate the changes of prevailing terms in the literature body over time. Meanwhile, the 10 most recurring and on average most cited author keywords are listed in Table 3. Consistently, medication errors were frequently listed and together with CPOE and ADE were associated with high citation rates of the respective manuscripts. Besides, publications concerning critical care, intensive care units, and children/pediatrics were also highly cited. To supplement these findings, the all-time top 10 most cited publications concerning medical errors are listed in Table 4. Half of them were published in the *Journal of the American Medical Association*.

**Table 2:** The 10 terms with the highest appearance (*n*) and citations per publication (CPP) for all-time and different time periods, respectively.

**Highest appearance**			**Highest citation**		
**Term**	***n* (%)**	**CPP**	**Term**	***n* (%)**	**CPP**
**All-time**					
Patient	5,371 (43.3)	20.1	Computerized physician order entry	174 (1.4)	65.8
Study	5,182 (41.7)	20.3	Patient day	133 (1.1)	62.8
Error	4,391 (35.4)	22.9	Medication order	203 (1.6)	45.0
Medication error	4,108 (33.1)	18.3	Inclusion criterion	157 (1.3)	42.9
Medical error	3,577 (28.8)	19.2	Preventability	150 (1.2)	42.7
System	3,343 (26.9)	21.3	MEDLINE	239 (1.9)	41.0
Hospital	2,956 (23.8)	21.9	Confidence interval	341 (2.7)	40.4
Analysis	2,790 (22.5)	19.1	Adverse drug event	628 (5.1)	37.7
Data	2,672 (21.5)	21.2	Burnout	214 (1.7)	36.2
Care	2,531 (20.4)	22.0	Injury	505 (4.1)	36.2
**1990s and before**					
Medication error	191 (53.4)	31.0	Month period	5 (1.4)	347.5
Error	115 (32.1)	72.6	Serious medication error	4 (1.1)	306.0
Patient	83 (23.2)	83.4	Patient day	8 (2.2)	297.9
Study	63 (17.6)	53.6	Decrease	9 (2.5)	288.0
Hospital	60 (16.8)	96.8	Hospitalization	5 (1.4)	274.2
System	51 (14.2)	75.3	Benefit	5 (1.4)	261.8
Medicine	48 (13.4)	56.5	Stage	5 (1.4)	255.7
Use	39 (10.9)	34.8	Participant	6 (1.7)	244.0
Drug	38 (10.6)	30.5	Study period	5 (1.4)	242.2
Medication	38 (10.6)	56.7	Medication error prevention	5 (1.4)	222.4
**2000s**					
Patient	1,398 (37.7)	43.8	Systematic review	56 (1.5)	127.3
Error	1,385 (37.4)	47.2	MEDLINE	46 (1.2)	125.7
Medical error	1,334 (36.0)	33.4	Preventability	46 (1.2)	107.6
Medication error	1,173 (31.7)	37.5	Confidence interval	99 (2.7)	104.7
Study	1,142 (30.8)	52.2	Computerized physician order entry	81 (2.2)	98.5
System	1,072 (28.9)	43.3	Odds ratio	52 (1.4)	94.0
Hospital	799 (21.6)	47.1	Researcher	61 (1.6)	91.2
Care	725 (19.6)	46.7	Patient day	49 (1.3)	89.3
Analysis	658 (17.8)	44.6	Hospital admission	45 (1.2)	87.4
Data	650 (17.5)	51.2	Study period	80 (2.2)	85.6
**Since 2010s**					
Study	3,876 (46.4)	11.1	Burnout	199 (2.4)	28.4
Patient	3,868 (46.3)	10.2	EMBASE	166 (2.0)	24.4
Error	2,907 (34.8)	9.6	Depression	99 (1.2)	21.7
Medication error	2,740 (32.8)	8.6	Symptom	165 (2.0)	21.0
Medical error	2,209 (26.4)	10.4	Primary outcome	112 (1.3)	20.9
Hospital	2,138 (25.6)	10.6	MEDLINE	193 (2.3)	20.8
System	2,117 (25.3)	9.3	CINAHL	103 (1.2)	20.6
Analysis	2,097 (25.1)	10.7	Inclusion criterion	135 (1.6)	20.5
Data	1,990 (23.8)	11.2	Degree	199 (2.4)	19.6
Care	1,780 (21.3)	11.3	Systematic review	311 (3.7)	19.5

**Table 3:** The 10 author keywords with highest appearance (*n*) and citations per publication (CPP) for all-time and different time periods, respectively.

**Highest appearance**			**Highest citation**		
**Keyword**	***n* (%)**	**CPP**	**Keyword**	***n* (%)**	**CPP**
**All-time**					
Patient safety	1,701 (13.7)	14.2	Errors, medication	75 (0.6)	38.1
Medication errors	1,401 (11.3)	14.2	CPOE	63 (0.5)	26.7
Medical errors	986 (7.9)	19.5	Hospitals	123 (1.0)	26.3
Medical error	650 (5.2)	15.5	Critical care	94 (0.8)	25.8
Medication error	606 (4.9)	11.6	Intensive care unit	83 (0.7)	24.7
Adverse events	278 (2.2)	22.3	Children	76 (0.6)	24.1
Safety	262 (2.1)	16.7	Quality of care	84 (0.7)	23.3
Medication safety	256 (2.1)	8.7	Quality assurance	123 (1.0)	22.6
Quality improvement	217 (1.7)	11.1	Adverse events	278 (2.2)	22.3
Nursing	195 (1.6)	11.9	Adverse drug event	74 (0.6)	21.5
**1990s and before**					
Medication errors	25 (7.0)	33.8	Prevention	2 (0.6)	93.5
Errors, medication	16 (4.5)	41.3	Medication error	7 (2.0)	90.4
Pharmacy, institutional, hospital	15 (4.2)	30.7	Databases	2 (0.6)	76.5
Administration	13 (3.6)	27.8	Nursing	2 (0.6)	65.5
Drug distribution systems	8 (2.2)	27.9	Prescription	2 (0.6)	65.5
Pharmacists, hospital	8 (2.2)	17.5	Nurses	3 (0.8)	62.0
Computers	7 (2.0)	41.3	Methodology	3 (0.8)	61.0
Medication error	7 (2.0)	90.4	Continuous quality improvement	2 (0.6)	58.5
Pharmaceutical services	7 (2.0)	29.9	Iatrogenic disease	3 (0.8)	57.7
Data collection	5 (1.4)	20.8	Pharmacist	2 (0.6)	57.0
**2000s**					
Patient safety	350 (9.4)	37.0	Critical care	20 (0.5)	77.1
Medication errors	318 (8.6)	35.8	Children	21 (0.6)	67.5
Medical errors	308 (8.3)	41.4	Pediatrics	45 (1.2)	50.6
Medical error	187 (5.0)	33.1	Emergency medicine	29 (0.8)	50.2
Medication error	146 (3.9)	24.0	Adverse events	77 (2.1)	48.0
Adverse events	77 (2.1)	48.0	Dosage	24 (0.6)	46.5
Safety	70 (1.9)	26.9	Ambulatory care	19 (0.5)	45.8
Hospitals	63 (1.7)	40.4	Errors, medication	43 (1.2)	45.4
Errors	57 (1.5)	34.5	Quality of care	25 (0.7)	44.6
Risk management	53 (1.4)	23.2	CPOE	27 (0.7)	43.9
**Since 2010s**					
Patient safety	1,351 (16.2)	8.3	Systematic review	54 (0.6)	22.3
Medication errors	1,058 (12.7)	7.2	Burnout	88 (1.1)	17.1
Medical errors	676 (8.1)	9.5	Quality assurance	67 (0.8)	15.4
Medical error	462 (5.5)	8.2	Quality of care	59 (0.7)	14.3
Medication error	453 (5.4)	6.4	Safety	192 (2.3)	12.9
Medication safety	229 (2.7)	6.2	Intensive care unit	67 (0.8)	12.8
Adverse events	201 (2.4)	12.5	Adverse events	201 (2.4)	12.5
Safety	192 (2.3)	12.9	Physicians	45 (0.5)	12.2
Quality improvement	183 (2.2)	7.5	Critical care	74 (0.9)	12.0
Nursing	160 (1.9)	9.3	CPOE	45 (0.5)	11.9

*CPOE, computerized physician order entry*.

**Table 4:** Top 10 most cited medical errors publications.

**Publication**	**Total citations**	**Citations per year**
Chaudhry, B., Wang, J., Wu, S., Maglione, M., Mojica, W., Roth, E., … & Shekelle, P. G. (2006). Systematic review: impact of health information technology on quality, efficiency, and costs of medical care. *Annals of Internal Medicine, 144*(10), 742-752.	1,615	115.4
Leape, L. L. (1994). Error in medicine. *JAMA, 272*(23), 1851-1857.	1,366	52.5
Bates, D. W., Leape, L. L., Cullen, D. J., Laird, N., Petersen, L. A., Teich, J. M., … & Vander Vliet, M. (1998). Effect of computerized physician order entry and a team intervention on prevention of serious medication errors. *JAMA, 280*(15), 1311-1316.	1,285	58.4
Koppel, R., Metlay, J. P., Cohen, A., Abaluck, B., Localio, A. R., Kimmel, S. E., & Strom, B. L. (2005). Role of computerized physician order entry systems in facilitating medication errors. *JAMA, 293*(10), 1197-1203.	1,237	82.5
Landrigan, C. P., Rothschild, J. M., Cronin, J. W., Kaushal, R., Burdick, E., Katz, J. T., … & Czeisler, C. A. (2004). Effect of reducing interns' work hours on serious medical errors in intensive care units. *New England Journal of Medicine, 351*(18), 1838-1848.	1,045	65.3
Shanafelt, T. D., Boone, S., Tan, L., Dyrbye, L. N., Sotile, W., Satele, D., … & Oreskovich, M. R. (2012). Burnout and satisfaction with work-life balance among US physicians relative to the general US population. *Archives of Internal Medicine, 172*(18), 1377-1385.	994	124.3
Kaushal, R., Bates, D. W., Landrigan, C., McKenna, K. J., Clapp, M. D., Federico, F., & Goldmann, D. A. (2001). Medication errors and adverse drug events in pediatric inpatients. *JAMA, 285*(16), 2114-2120.	990	52.1
Gurwitz, J. H., Field, T. S., Harrold, L. R., Rothschild, J., Debellis, K., Seger, A. C., … & Bates, D. W. (2003). Incidence and preventability of adverse drug events among older persons in the ambulatory setting. *JAMA, 289*(9), 1107-1116.	957	56.3
Ash, J. S., Berg, M., & Coiera, E. (2004). Some unintended consequences of information technology in health care: the nature of patient care information system-related errors. *Journal of the American Medical Informatics Association, 11*(2), 104-112.	894	55.9
Bates, D. W., & Gawande, A. A. (2003). Improving safety with information technology. *New England Journal of Medicine, 348*(25), 2526-2534.	783	46.1

*Citations per year were calculated as total citations/number of years since publication (i.e., 2020 minus year of publication)*.

## Discussion

In this work, we have identified and bibliometrically analyzed 12,415 publications concerning medical errors.

The performed temporal publication number analysis (Figure 1) revealed steady growth of the body of literature dealing with medical errors, identifying this research area to be of increasing scientific interest. The trend line of publication counts plotted against time (Figure 1) reveals that the numbers of publications in this area increased linearly with a slower rate until 1999, exponentially increased in the period 2000–2010, and again went into steady but more rapid linear-increase mode since 2010. The exponential increase in publications in the decade following 1999 is likely to be triggered and highly influenced by the landmark report *To Err Is Human* published by the Institute of Medicine in 1999 (10, 11). The growth rate of medical errors literature (Figure 1) is faster than that of another recently analyzed scientific area with perceived high importance, food toxicology (12), and resembles the growth rate identified to occur with antioxidant research literature (13). Mirroring the three time periods of different growth rate of the literature body on medical errors (as identified in the trend line presented in Figure 1), further analysis was focused on comparison of features of the literature subsets published in the three respective periods: (i) the 1990s and before; (ii) the 2000s, and (iii) since the 2010s.

In respect to the type of publication (Figure 2), we noted that 70.9% of the total publications were original research articles, whereby the temporal analysis in the three time periods revealed an increasing share of original research articles with 55.9% in the 1990s and before, 68.7% in the 2000s, and 72.4% since the 2010s. Despite this trend indicating increasing amount of original research in the scientific area of medical errors, the total share of 70.9% is still a bit lower than the original research article shares of other recently analyzed biomedical scientific fields such as neuropharmacology (original research articles share of 72.3%) (14), biotechnology (73.2%) (7), and ethnopharmacology (84.6%) (15). Probably this lower share of original research articles is due to the intrinsic difficulties associated with the medical errors field of research (e.g., difficulties in reliable identification of medical errors, differences and discrepancies in definitions and used terminology, and diverse complicating societal, legal, ethical, economical, and behavioral aspects) (16–18).

Concerning authorship of the medical error literature, examination of the all-time top 10 list presented in Table 1 reveals that the most productive author by far (182 publications, almost 3-fold more than the second author on the list) was David W. Bates. Professor Bates also was the leader concerning H index of the analyzed medical error publications (H index = 61) and had the second highest CPP (CPP = 96.1) among the top 10 most productive authors. Professor Bates has made major contributions in the area of application of information technology to patient safety, outcome assessment, and quality of care, including CPOE implementation (19). Remarkably, around half (46.4%) of all medical error publications were affiliated with the United States (being also the home country of Professor Bates), where 8 out of the top 10 most productive institutions were located (Table 1). While the United States is clearly one of the leading countries in terms of scientific productivity in many different research areas, its publication share (46.4%) in the medical errors literature is clearly higher than the shares of publications affiliated with the United States in other scientific areas with medical relevance such as Alzheimer disease (39.4%) (20), bariatric surgery (39.3%) (21), and neuropharmacology (38.0%) (14).

In respect to commonly discussed and highly cited topics in the medical errors literature (Table 2), prevailing themes are revealed by the common use/citation rate of terms related to drug administration (“medication error,” “medication error prevention,” “medication,” “serious medication error,” “medication order,” “drug,” “adverse drug event”), mental conditions (in the healthcare professionals or the affected patients) that might be associated with medical errors (“depression,” “burnout”), and medical information technology (“CPOE”; terms associated with medical literature databases: “EMBASE,” “MEDLINE,” “CINAHL”). The high percentage of publications referencing medications and associated terminology is in line with the known high share (25%) of errors related to drugs as a source of preventable patient harm (2). Analysis of the bubble maps (Figure 4) of the prevailing terms in the three analyzed periods also indicate diversification of research topics (increased number of bubbles) with the progression of time. This observation can be linked with the higher share of publications related to “medication error” in the first analyzed time period (53.4%, representation of the term by the biggest bubble in the graph depicting the time period “1990s and before”). While in the next two periods the share of publications referencing “medication error” decreased (to 31.7 and 32.8%, respectively), many new term-bubbles appeared, supporting the noted diversification of research themes. This shift associated with a decreased share of medication error-related publications might also be the reason for the decreasing share of articles published in journals of the category “pharmacology pharmacy” (Figure 3).

Analysis of publication keywords listed by the authors (Table 3) confirms the prevalence and importance of themes related to medications (“medication error,” “medication errors,” “adverse drug event,” etc.), mental health conditions (“burnout”), and digital health technology (“CPOE,” “computers,” “databases,” “CPOE”). The known high share of medical errors in advanced specialties (e.g., intensive care) (2) is also consistent with the high citation rate and prevalence of keywords such as “critical care” (CPP = 25.8; keyword listed in 0.8% of all analyzed publications) and “intensive care unit” (CPP = 24.7; listed in 0.7% of all analyzed publications). Interestingly, publications having a keyword “children” were also among the highest cited (CPP = 24.1). The high importance (reflected by high citation rate) of literature dealing with medical errors in children becomes evident when taking into consideration that there is much less research studying medical errors and preventable harms in children than in adults (2), but the rates of preventable ADE and potential ADE in pediatric inpatients might be in the same range or even higher than in adults (22–24).

The characteristics of the top 10 most cited publications (Table 4) (22, 25–33) provide further affirmations and rationalizations that can be linked to some of the major findings from the analysis of the entire medical errors publications set: Professor Bates, the most productive author, was in the author list of half of the 10 most cited publications (22, 27, 29, 31, 33), and a significant share of this 10 publications was focused on topics identified as highly impactful/prevailing, including applications of medical information technology/CPOE (25, 27, 28, 32, 33), and medical errors related to medications/ADE (22, 27, 28, 31), intensive care units (29), children (22), or burnout among physicians (30).

## Limitations

The search strategy utilized in this work identifies a well-defined literature set referring to medical errors in the WoS database. However, it should be noted that relevant articles only containing different terms of relevance, for example, “surgery errors” or “diagnostic mistakes,” are not identified by our approach and are therefore not included in this analysis. We did not include word combinations other than medical/healthcare errors/ mistakes (and variations/combinations thereof) in the search strategy in order to not introduce bias in our study. For example, adding “surgery errors” to the search strategy would have yielded additional relevant articles, but at the same time would have resulted in the specific enrichment of the yielded literature set with surgery-related articles. This would have prevented an unbiased estimation of the prevalence of the theme “surgery” within the medical errors literature. Moreover, relevant scientific articles from emerging journals that are not yet indexed in WoS are also not covered in our analysis. Merging of citation data originating from different databases cannot be done unbiasedly because each database collects citation counts differently. Web of Science was chosen for an unbiased total-scale analysis of the literature on medical errors as it represents the most referred and qualitatively reliable database of scientific literature, which is also used as a basis for the most established calculation of journal impact factors [Journal Citation Reports (JCR)]. Finally, it should be noted that while this work represents analysis of the global scientific literature, with the United States contributing to around half of all publications and with the following three highest-contributing countries also being first-world and English-speaking (England, Canada, Australia), the analysis outcomes are heavily influenced by this subset of the global population.

## Conclusions

The analysis of literature concerning medical errors indicates that applications related to digital health technology (e.g., CPOE), and errors related to drugs/medications/ADE, to mental conditions (burnout, depression) in healthcare professionals associated with medical errors, to advanced specialties (e.g., intensive care), and to children/pediatrics, represent themes of leading importance. Consequently, of especially high impact might be future research on the interface of several of these prevailing themes (e.g., the application of digital applications to monitor the mental health status of healthcare professionals in pediatric intensive care units).

## Data Availability Statement

All datasets generated in this study are included in the article/supplementary material.

## Author Contributions

AA, AY, MK-P, and HW: conceived and designed the study. AY: extracted and analyzed the data. AA and AY: drafted the initial manuscript draft. AA, AY, EK, FE, ES, MK-P, and HW: critically revised the manuscript, interpreted data, and approved the final manuscript. All authors contributed to the article and approved the submitted version.

## Conflict of Interest

The authors declare that the research was conducted in the absence of any commercial or financial relationships that could be construed as a potential conflict of interest.
